# Supplementary material for: The recombination landscape of the barn owl, from families to populations
Source: Genetics. 2024 Nov 15;229(1):iyae190. doi: 10.1093/genetics/iyae190 (PMC11708917; doi:10.1093/genetics/iyae190)
Supplement: iyae190_Supplementary_Data [file iyae190_supplementary_data.zip › File S1 - iyae190.pdf]

Supplementary Material

Supplementary Text **1**
1 - Making a linkage map 1
2. Annotating Centromeres 3
Bibliography 4
Supplementary Tables **5**
Supplementary Figures **8**

Supplementary Text

1 - Making a linkage map

We used *SeparateChromosomes* in LepMap3 (Rastas, 2017) to identify the putative linkage groups (LGs) based on a user-defined logarithm of odds (LOD) score cutoff. The choice of the correct LOD score for each dataset will depend on the family structure. The developer of LepMap3 suggests the use of a LOD score that gives a number and size of linkage groups (LGs) that match the known chromosome count for the species. This is complicated when one does not know how many (and how much of each) chromosomes of the species are actually included in the genome assembly. Additionally, in studies with many markers and due to the dubious nature of some parts of the genome assembly (Ducrest et al., 2020; Machado et al., 2022) the distribution of linkage group sizes might deviate from the expectation. Here we try to explain and justify our choice of the "correct" LOD score. In classical linkage analysis of families the usual LOD cutoff is 3 meaning a 1000 to 1 chance of the markers being linked compared to un-linked. If the LOD score chosen is less than 3 then markers that appear linked might in fact belong to different chromosomes and merging of chromosomes into the same LG will be observed. On the contrary if the cutoff is too high then only strongly linked markers will appear to be belonging to the same linkage group meaning that the user will observe over-splitting of LGs. The approach we used to identify

the "correct" LOD score is based on the above expectation, an assumption that our reference genome had confidently placed the largest scaffolds together and the expectation that a small number of markers will be misplaced. A similar approach was used in (Peñalba et al., 2020).

Specifically, we ran the *SeparateChromosomes* module for different LOD scores from 11 to 21. We plotted the distribution of markers per linkage group and we compared it to the actual distribution of markers in super-scaffolds (Figure S1 in this document). We then identified a LOD of 15 as the balanced LOD score between overmerging and a distribution that matched our expectations. We compared the results obtained with a LOD score of 15 to the results with LOD score 16 and 17. Since the lower the LOD score, the more likely the overmerging, LOD 15 should exhibit the largest amount of merging between the three. Thus we manually checked the LGs that contained more than one super-scaffold. We found most merges included one marker from multiple scaffolds and a large number of markers from another one. We assumed that the single markers added were erroneously placed and removed them from that LG. Only one split and two scaffold merges were supported by enough markers.

The split of Super-Scaffold_2 into linkage group 1 and linkage group 24 was further validated by looking into LepMap3 runs with smaller LOD cutoffs (where overmerging is expected). Because the split was present in LOD 13 we considered it well supported. The scaffolding of the 2 parts of Super-Scaffold_2 was performed with bionano optical mapping with the addition of a long sequence of N nucleotides (Machado et al., 2022).

One merge was that of Super-Scaffold_100000100064 and Super-Scaffold_200000178 in linkage group 41 but both scaffolds have very few markers and LepMap3 could not sufficiently order this linkage group so we discarded it.

The other merge was that of Super-Scaffold_3 and Super-Scaffold_49 in linkage group 20. This was verified by looking for this merge in datasets with a larger LOD score cutoff (where over splitting is expected). The merge appeared up to LOD 18. Synteny analysis showed the two scaffolds mapping side to side on chromosome 12 of the chicken (Figure S4 in this

document). Using the less stringently filtered set of markers and a LOD5 cutoff we manually oriented the linkage group 20 for downstream analyses.

The Z chromosome was identified in previous studies as Super-Scaffold_13 and Super-Scaffold_42 (Machado et al., 2022, Cumer et al., 2023). The linkage map of the Z chromosome (linkage group 40) was built manually by using the LOD5 dataset.

2. Annotating Centromeres

To annotate centromeric positions we used RepeatObserver (Elphinstone et al., 2023) and TRASH (Wlodzimierz et al., 2023). RepeatObserver uses the repeat density along each ‘chromosome’ and identifies the lowest dip in repeat density as a putative centromere while TRASH annotates tandem repeats along the sequence. Both software depend on the repeat structure of centromeric and pericentromeric sequences to annotate such elements. We used a combination of both in a version of the barn owl assembly v. 4.0 (Machado et al., 2022) where we removed ‘N’ nucleotides. We used both software with default parameters and then used annotations of centromeres from RepeatObserver in the 250kb and 500kb resolution and overlapped those with annotated tandem repeats from TRASH (Figure S7 in this document). Because all autosomes of the barn owl are telocentric or acrocentric we disregarded annotations where the centromere was not placed in the most distal 20% of the sequence. This left us with 18 linkage groups where the centromere was annotated.

One of the most pronounced effects of a centromere on recombination rates is its localised suppression (Haenel et al., 2018; Peñalba & Wolf, 2020). However, when we looked at the recombination rate close to the annotated centromeres we often saw a peak instead of a dip (Figure S8 in this document). We believe this is because the telomeres can also harbour tandem repeats and be mistakenly annotated as a centromere. Coupled with the fact that usually telomeres show an increase of recombination we have reason to believe that this is the case.

Bibliography

Ducrest, A.-L., Neuenschwander, S., Schmid-Siegert, E., Pagni, M., Train, C., Dylus, D., Nevers, Y., Vesztrocy, A. W., San-Jose, L. M., Dupasquier, M., Dessimoz, C., Xenarios, I., Roulin, A., & Goudet, J. (2020). New genome assembly of the barn owl (Tyto alba alba). *Ecology and Evolution*, *10*(5), 2284–2298. https://doi.org/10.1002/ece3.5991

Elphinstone, C., Elphinstone, R., Todesco, M., & Rieseberg, L. (2023). *RepeatOBserver: Tandem repeat visualization and centromere detection*. https://doi.org/10.1101/2023.12.30.573697

Haenel, Q., Laurentino, T. G., Roesti, M., & Berner, D. (2018). Meta-analysis of chromosome-scale crossover rate variation in eukaryotes and its significance to evolutionary genomics. *Molecular Ecology*, *27*(11), 2477–2497. https://doi.org/10.1111/mec.14699

Machado, A. P., Cumer, T., Iseli, C., Beaudoing, E., Ducrest, A.-L., Dupasquier, M., Guex, N., Dichmann, K., Lourenço, R., Lusby, J., Martens, H.-D., Prévost, L., Ramsden, D., Roulin, A., & Goudet, J. (2022). Unexpected post-glacial colonisation route explains the white colour of barn owls (Tyto alba) from the British Isles. *Molecular Ecology*, *31*(2), 482–497. https://doi.org/10.1111/mec.16250

Peñalba, J. V., Deng, Y., Fang, Q., Joseph, L., Moritz, C., & Cockburn, A. (2020). Genome of an iconic Australian bird: High-quality assembly and linkage map of the superb fairy-wren (Malurus cyaneus). *Molecular Ecology Resources*, *20*(2), 560–578. https://doi.org/10.1111/1755-0998.13124

Peñalba, J. V., & Wolf, J. B. W. (2020). From molecules to populations: Appreciating and estimating recombination rate variation. *Nature Reviews Genetics*, *21*(8), 476–492. https://doi.org/10.1038/s41576-020-0240-1

Rastas, P. (2017). Lep-MAP3: Robust linkage mapping even for low-coverage whole genome sequencing data. *Bioinformatics*, *33*(23), 3726–3732.

https://doi.org/10.1093/bioinformatics/btx494

Wlodzimierz, P., Hong, M., & Henderson, I. R. (2023). TRASH: Tandem Repeat Annotation and Structural Hierarchy. *Bioinformatics*, *39*(5), btad308. https://doi.org/10.1093/bioinformatics/btad308

Supplementary Tables

Table S1. Number of SNPs that passed filters for each population and for each analysis.
Abbreviations: CH - Switzerland full dataset, GB - Great Britain, PT - Portugal, CH13 - first subset of 13 individuals from Switzerland, CH13_[2,3,4,5] - further subsets of 13 individuals from Switzerland. Mac - minor allele count, π- nucleotide diversity

**Population**	**Sample size**	**SMC++** 10% missingness HWE, 5%MAF	**Pyrho** 10% missingness, 10bp distance, HWE, 5% MAF	**π** 50% missingness, 5 mac
CH	76	6,501,194	5,814,488	8,512,324
GB	13	5,178,978	4,756,461	3,211,420
PT	13	6,931,299	6,176,928	3,325,626
CH13	13	6,242,237	5,621,093	-
CH13_2	13	6,256,154	5,587,278	-
CH13_3	13	5,979,331	5,446,160	-
CH13_4	13	6,297,860	5,766,358	-
CH13_5	13	5,767,988	5,291,416	-

Table S2. **Hyperparameters used in pyrho.**
Abbreviations: CH - Switzerland full dataset, GB - Great Britain, PT - Portugal, CH13 - first subset of 13 individuals from Switzerland, CH13_[2,3,4,5] - further subsets of 13 individuals from Switzerland.

**Population**	**Window Size**	**Block penalty**
CH	40	15
GB	30	15
PT	40	15
CH13	40	15
CH13_2	30	15

CH13_3	30	15
CH13_4	40	15
CH13_5	60	15

Table S3. Table of linkage groups in the barn owl assembly.
Synteny based on highest matching proportions in Figure S4.

Linkage Group	Super-Scaffold	Male cM	Female cM	Sex-averag e cM	Length	SNPs	Male Crossover s	Female Crossover s	Chicken Synteny
1	Super-Scaffold_2	94.107	74.808	83.651	62997631	233957	123	105	Chr01A
2	Super-Scaffold_14	87.053	88.278	86.7395	60520781	275476	121	132	Chr01C
3	Super-Scaffold_6	95.227	98.167	96.697	56534680	226198	134	133	Chr04C
4	Super-Scaffold_40	79.36	69.653	73.3955	51327406	199776	121	119	Chr03C
5	Super-Scaffold_9	69.405	63.161	66.283	43766637	172811	124	119	Chr02E
6	Super-Scaffold_38	64.885	68.771	66.828	42002052	158486	95	101	Chr02A
7	Super-Scaffold_1	73.098	85.345	76.994	41834277	160969	128	134	Chr06
8	Super-Scaffold_7	65.329	73.471	67.6605	44054830	172361	113	127	Chr07
9	Super-Scaffold_18	56.309	91.277	70.3775	35172814	148801	118	143	Chr05B
10	Super-Scaffold_16	71.966	81.806	76.886	34944653	147227	112	133	Chr03A
11	Super-Scaffold_22	59.67	55.882	57.0815	41455493	175289	89	90	Chr03B
12	Super-Scaffold_45	66.729	80.751	73.74	42642627	150204	89	111	Chr01D
13	Super-Scaffold_27	41.611	38.413	40.012	33925083	136664	54	53	Chr02D
14	Super-Scaffold_10 00006	92.275	94.986	92.9455	36032128	133280	122	128	Chr08
15	Super-Scaffold_10	48.976	66.663	57.8195	29985952	119496	81	96	Chr09
16	Super-Scaffold_21	62.717	63.426	63.0715	23061809	108219	84	85	Chr01B
17	Super-Scaffold_48	72.301	71.875	69.2295	28624032	124717	97	126	Chr02C
18	Super-Scaffold_23	48.154	53.41	49.208	30848580	120197	71	101	Chr04B
19	Super-Scaffold_26	72.899	59.702	64.8665	24009840	83606	117	126	Chr11
20	Super-Scaffold_3	66.704	61.351	63.6575	20937722	93166	111	125	Chr12
20	Super-Scaffold_49	8.934	13.413	11.1735	4323735	12347	11	8	Chr12
21	Super-Scaffold_17	58.995	67.726	62.99	23302321	93559	81	101	Chr13
22	Super-Scaffold_8	57.406	71.539	63.397	25136324	80333	95	105	Chr05A
23	Super-Scaffold_5	57.736	73.575	65.6555	24525012	98233	97	120	Chr04A
24	Super-Scaffold_2.2	59.771	66.141	62.956	28222627	110122	82	93	Chr01E
25	Super-Scaffold_28	72.05	70.696	70.48	21137564	72500	105	97	Chr14
26	Super-Scaffold_46	72.407	45.927	56.6875	18736733	95000	107	100	Chr02B
27	Super-Scaffold_41	69.437	67.062	68.2495	24601080	83599	96	99	Chr10
28	Super-Scaffold_33	66.793	68.475	67.1625	18160456	75250	103	101	Chr20
29	Super-Scaffold_29	63.754	74.699	69.2265	14846335	72091	89	103	Chr05C
30	Super-Scaffold_11	55.043	49.746	52.3945	13317354	64432	113	99	Chr17

31	Super-Scaffold_44	29.641	40.023	34.832	17163611	65173	40	54	Chr15
32	Super-Scaffold_39	63.385	60.885	61.28	15349174	63829	88	90	Chr18
33	Super-Scaffold_19	72.995	90.896	77.616	13532077	65036	130	128	Chr19
34	Super-Scaffold_20000042	61.251	69.911	64.646	16068779	63475	90	99	Chr21
35	Super-Scaffold_20	65.205	46.177	55.1755	11375373	46410	95	92	Chr23
36	Super-Scaffold_30	48.149	58.798	50.094	7860098	42885	112	116	Chr26
37	Super-Scaffold_32	58.658	55.109	55.422	9466668	38944	77	87	Chr24
38	Super-Scaffold_12	45.774	64.127	54.144	7647940	32695	68	99	Chr27
39	Super-Scaffold_34	50.345	68.177	59.261	6755002	31818	87	101	Chr22
40	Super-Scaffold_13	23.788	0	11.894	48838493	235	42	0	ChrZ
40	Super-Scaffold_42	69.28	52.728	61.004	41426968	10316	102	71	ChrZ

Supplementary Figures

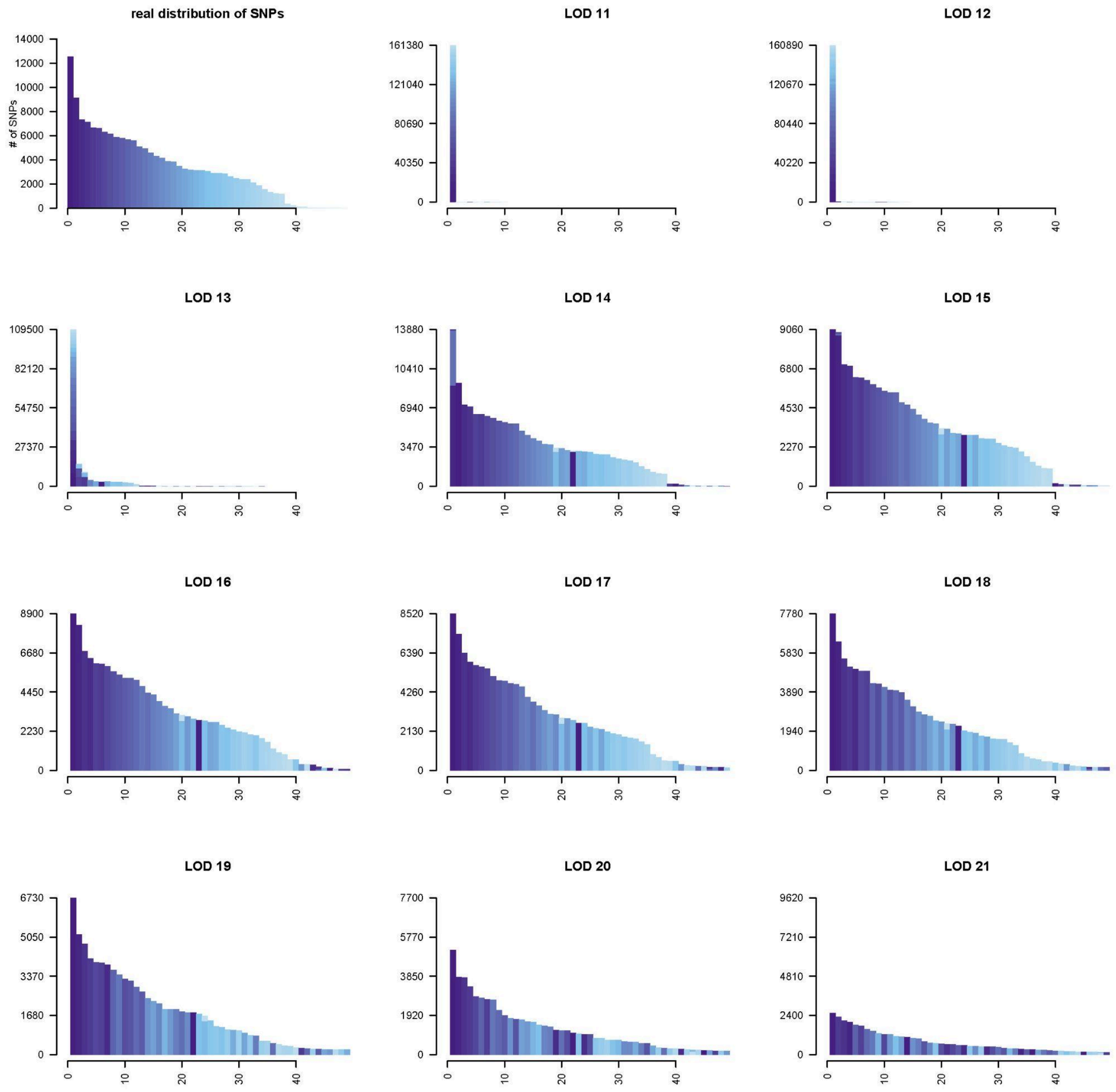

Figure S1. Linkage group length (in # of SNPs) for each LOD score cutoff in LepMap3's SeparateChromosome output.

Y-axis shows the number of SNPs and x axis the number (order) of linkage groups. Top-left panel is the observed distribution of SNPs in the assembly scaffolds. Scaffolds are coloured based on their physical length (darker = longer). Note how LOD15 (2nd row, 3rd column) shows a similar profile with the observed scaffolds except for the splitting of the longest scaffold into 2 linkage groups (a split observed in LOD14 too - as a dark line between marks 20 and 30 on the x-axis). The one merged linkage group can be seen around mark 20 on the LOD15 plot (a bar with 2 colours. We are confident in this merge since it appears for larger LOD scores where over splitting would be expected.

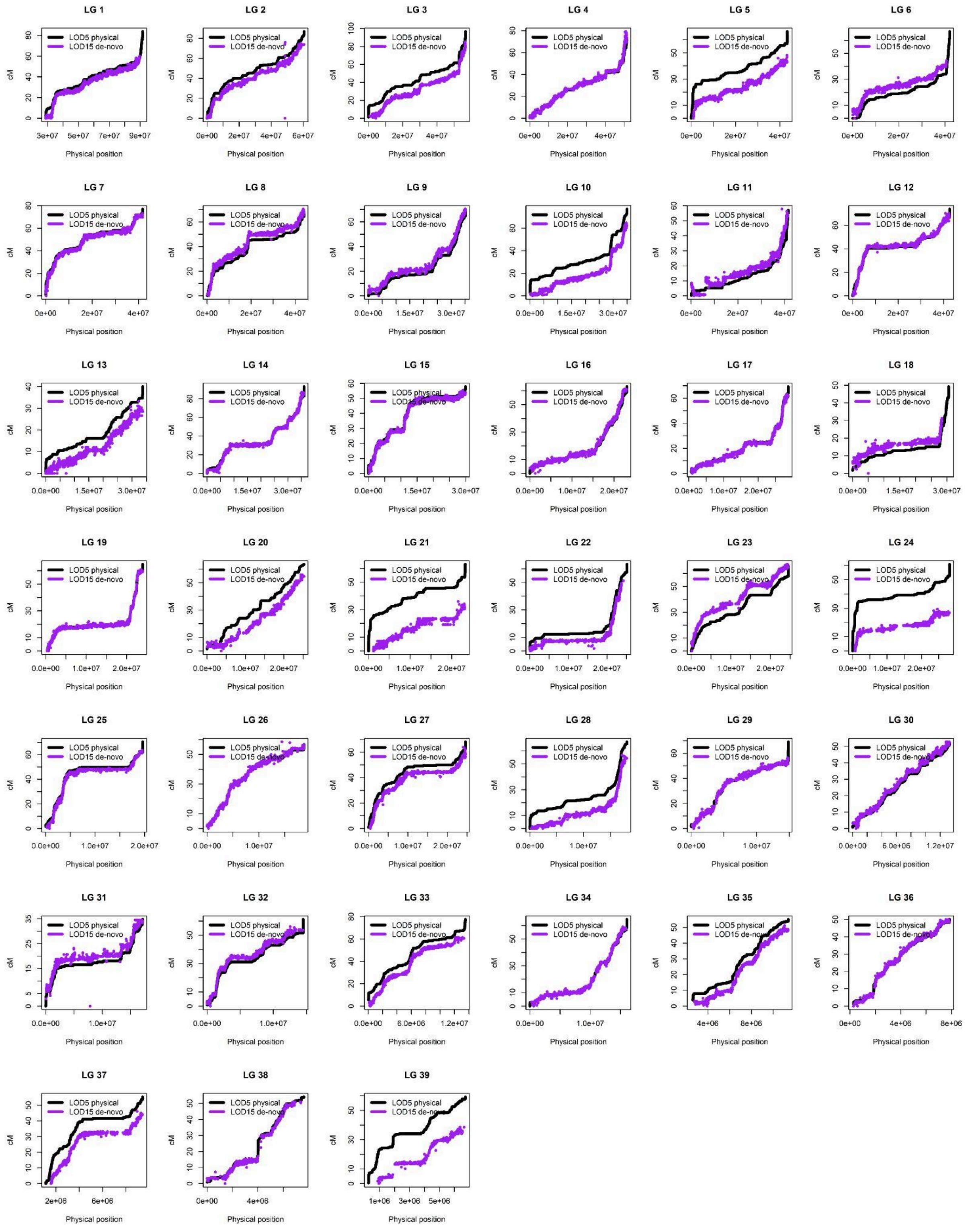

Figure S2. Comparison of LOD15 de novo ordering and LOD5 physical order linkage maps.

Plots show genetic map positions (cM against physical positions (bp) for all linkage groups except 20 and 40. Purple points show the de-novo ordering of 163,950 SNPs and black points show the full final map with 4,889,667 SNPs while enforcing physical positions in the ordering step.

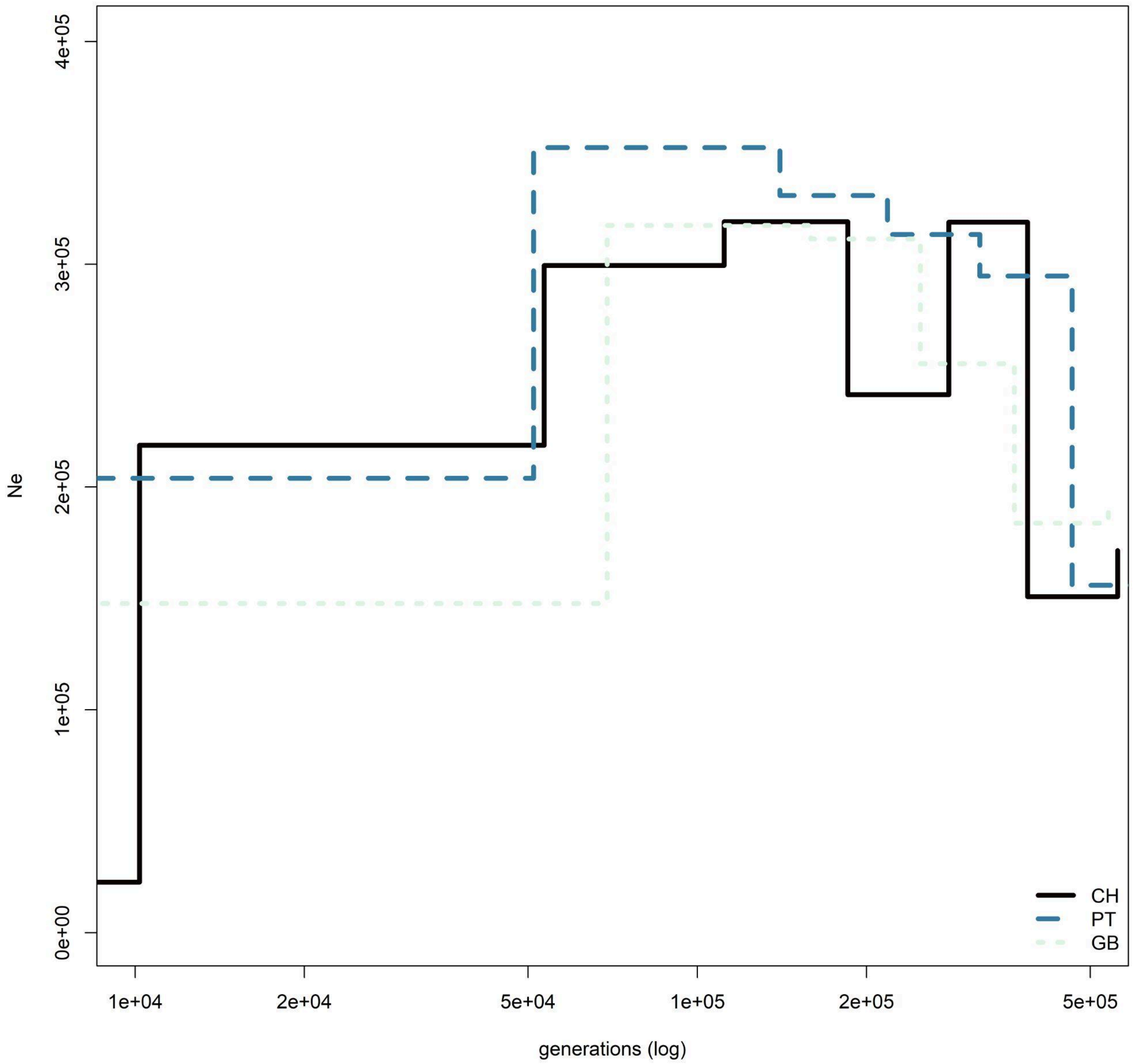

Figure S3. Inferred effective population sizes (Ne) from SMC++ for each dataset. Abbreviations: CH - Switzerland full dataset, GB - Great Britain, PT - Portugal. Mutation rate used to scale the y-axis was 1.93e-9 from Bergeron et al., 2023 estimated for the snowy owl (*Bubo scandiacus*).

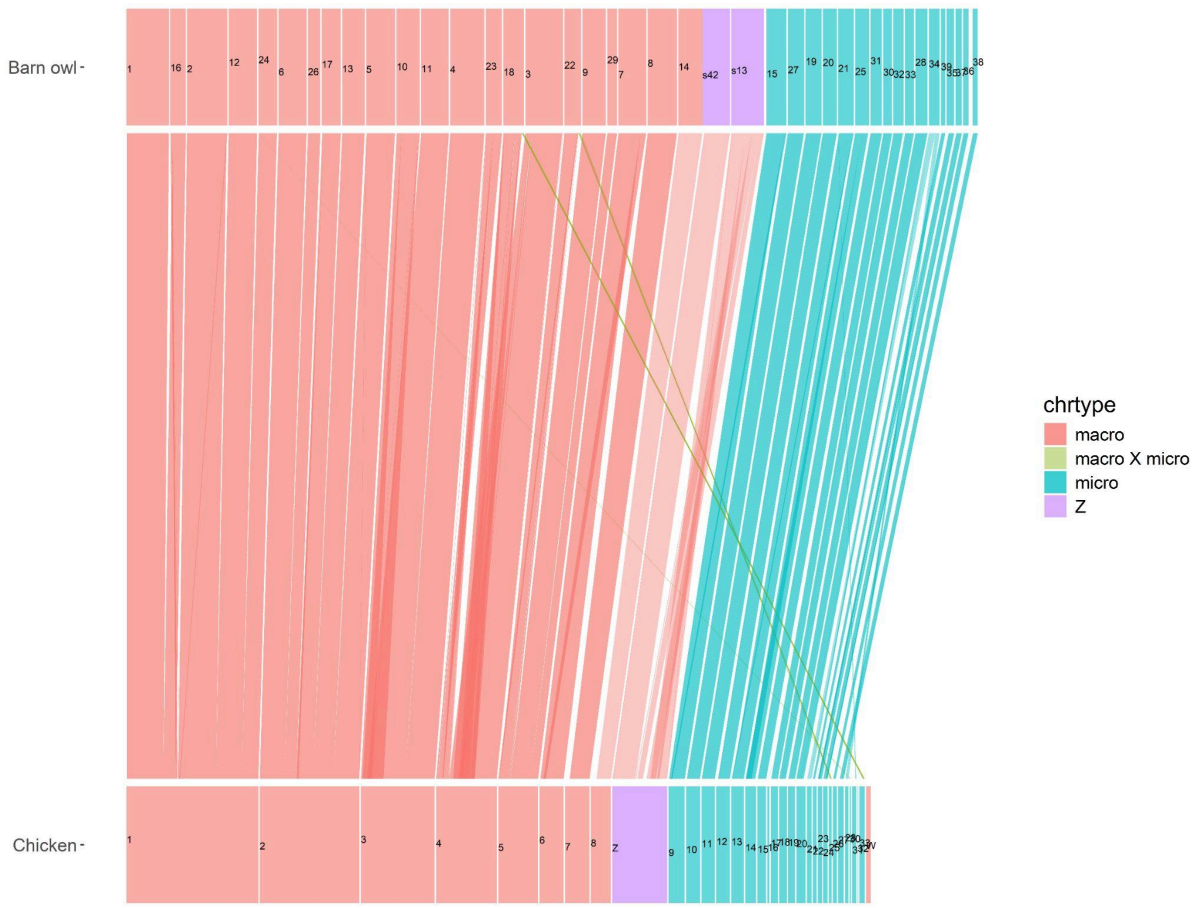

Figure S4. Synteny plot of barn owl (*Tyto alba*) linkage groups to chicken (*Gallus gallus*) chromosomes.

Top row is barn owl linkage group number ordered to match the chicken synteny (ordering based on last column of Table S3). Synteny is produced by mapping barn owl linkage groups to chicken chromosomes. Colours correspond to chromosome types, with the sex chromosome (Z) in purple. Note that super-scaffolds 13 and 42 correspond to LG40,(i.e. the Z chromosome) but they are represented here separately. The orientation of the barn owl linkage groups is inverted to match chicken chromosomes.

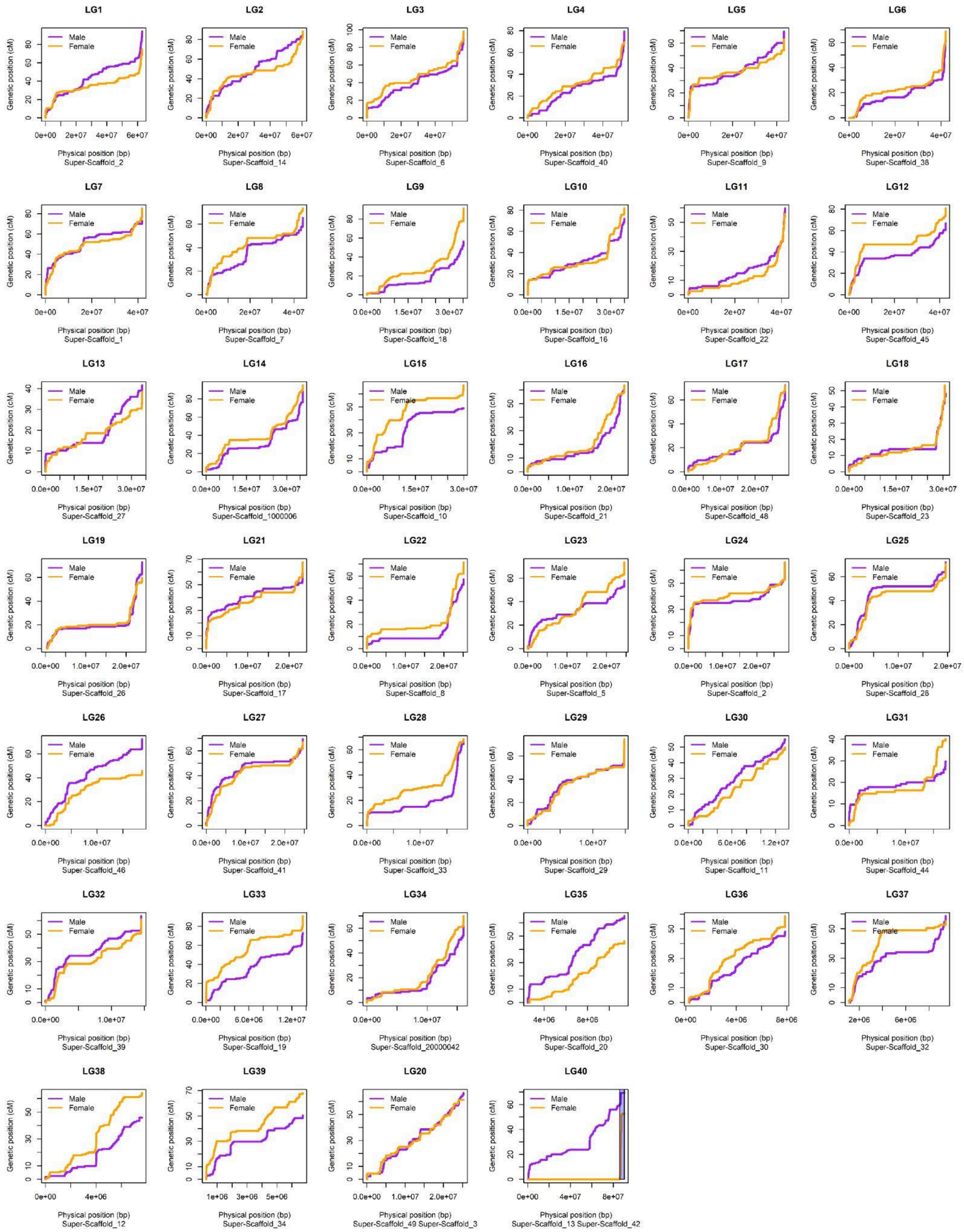

Figure S5. Sex-specific Marey maps.

On the y-axis is the cumulative cM position and on the x-axis the physical positions.For the last plot the blue shaded region is the pseudoautosomal region

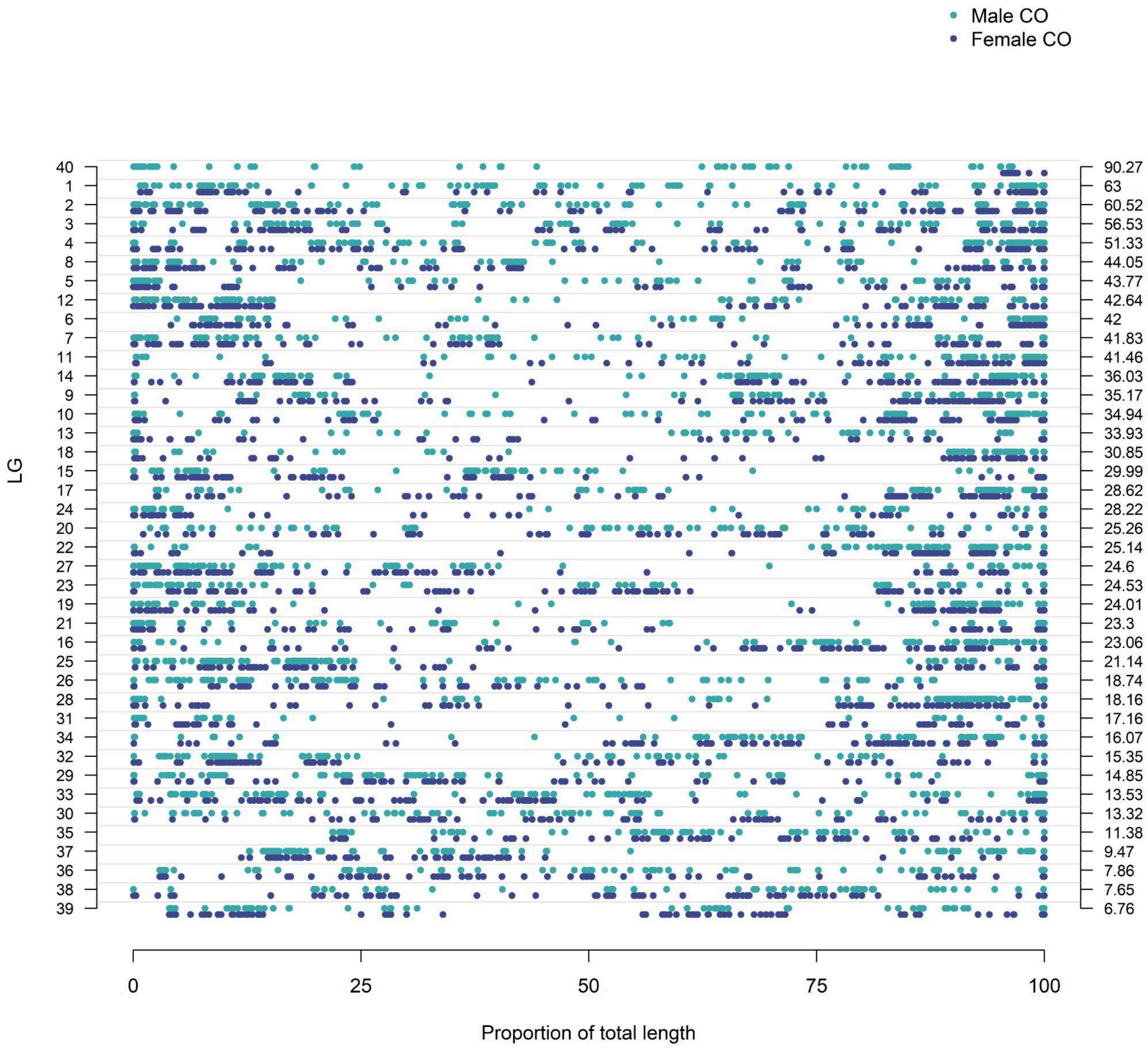

Figure S6. LepMAP3 inferred crossovers

Male (green) and Female (blue) crossovers along the length of all linkage groups (ordered by size with larger on top). Axis on the right is length in Mb.

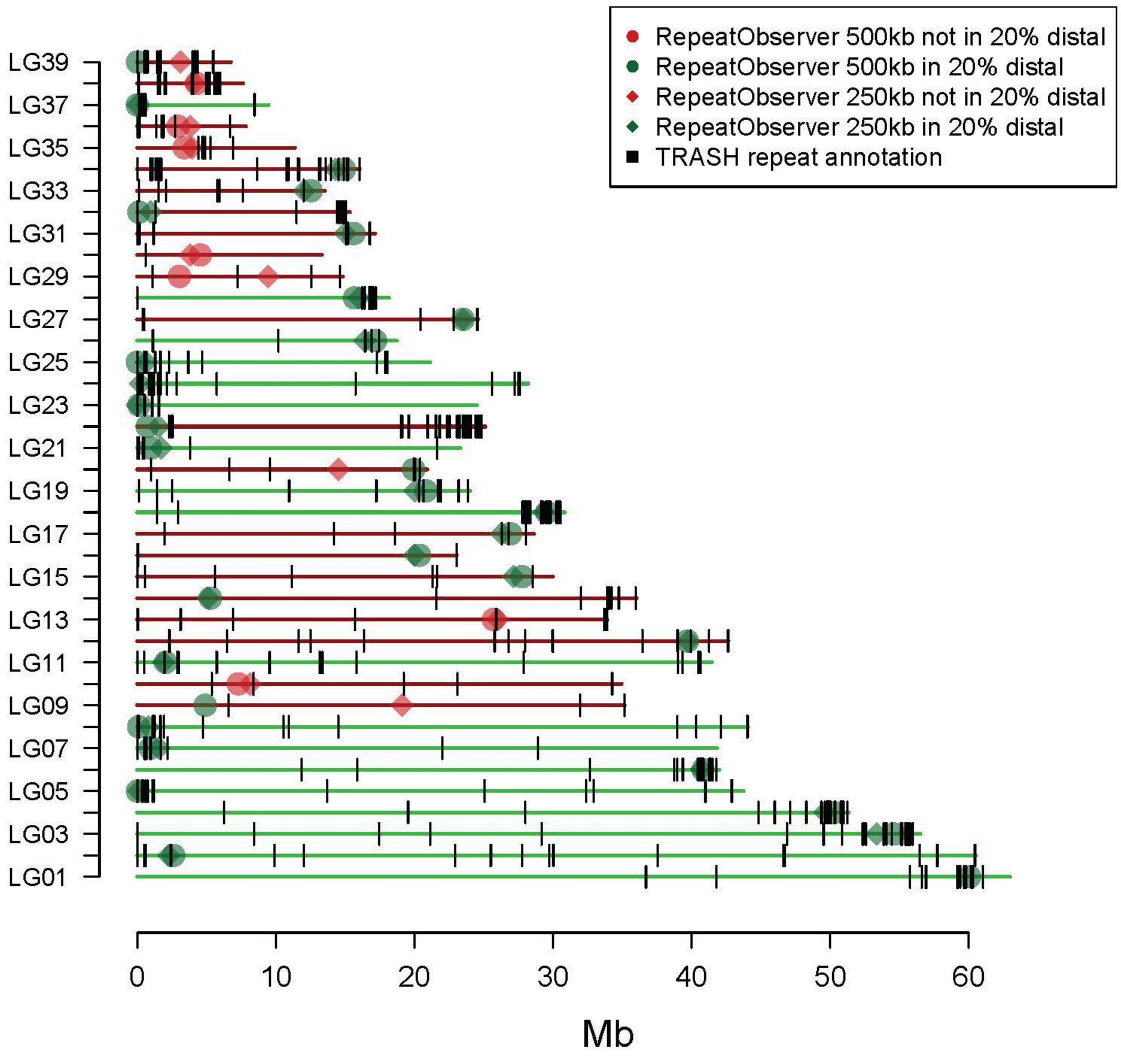

Figure S7. Centromere annotation of barn owl linkage groups.

Each line is a linkage group with annotated centromeres from RepeatObserver and annotated tandem repeats. Green lines are the annotations we manually considered good enough to be considered.

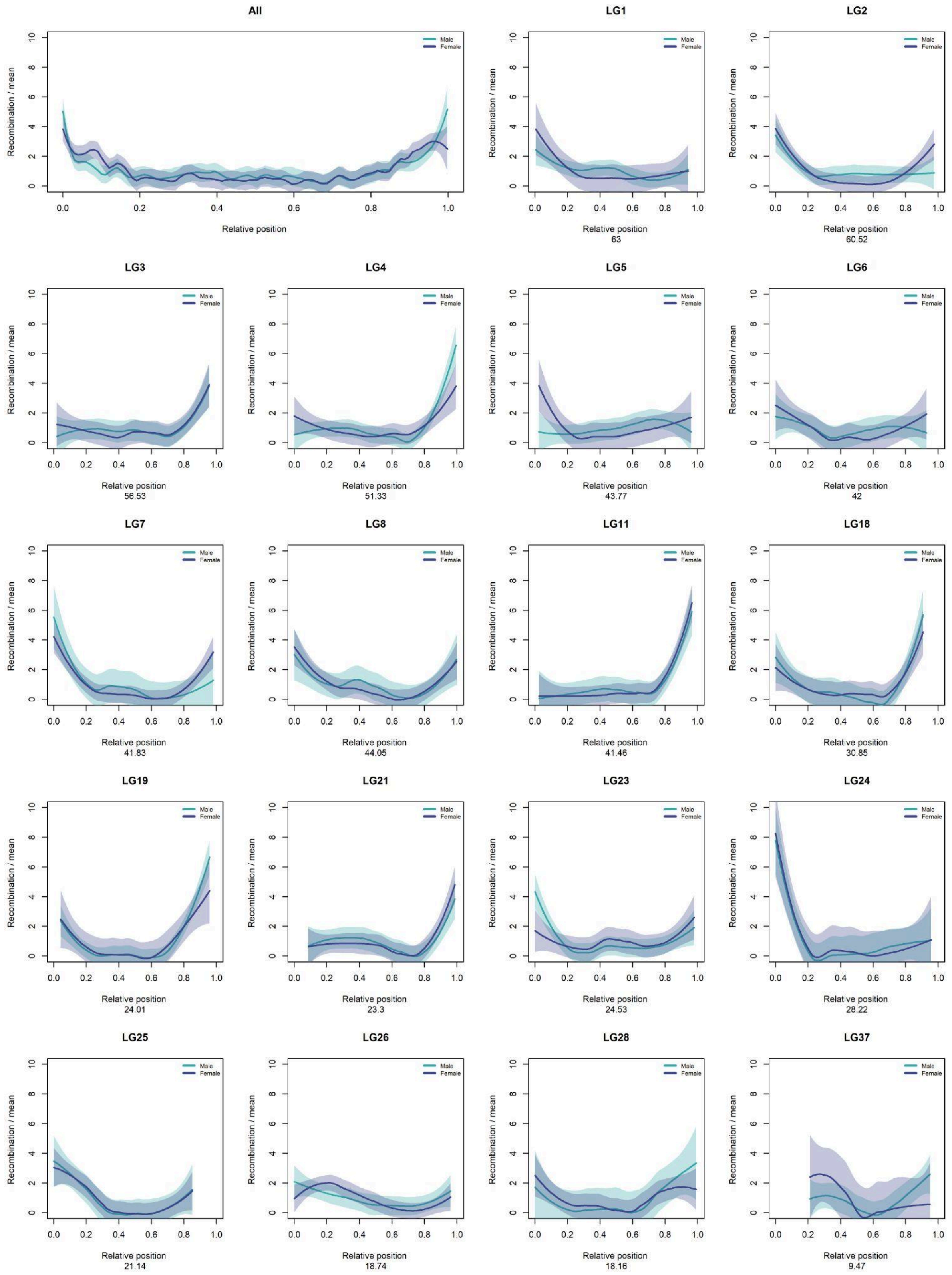

Figure S8. Sex-specific recombination relative to putative centromeric position.
First plot in top row is the cumulative local polynomial regression across all linkage groups. Further plots are for each linkage group where a centromere was annotated. The x-axis starts from the centromeric end of the linkage group.

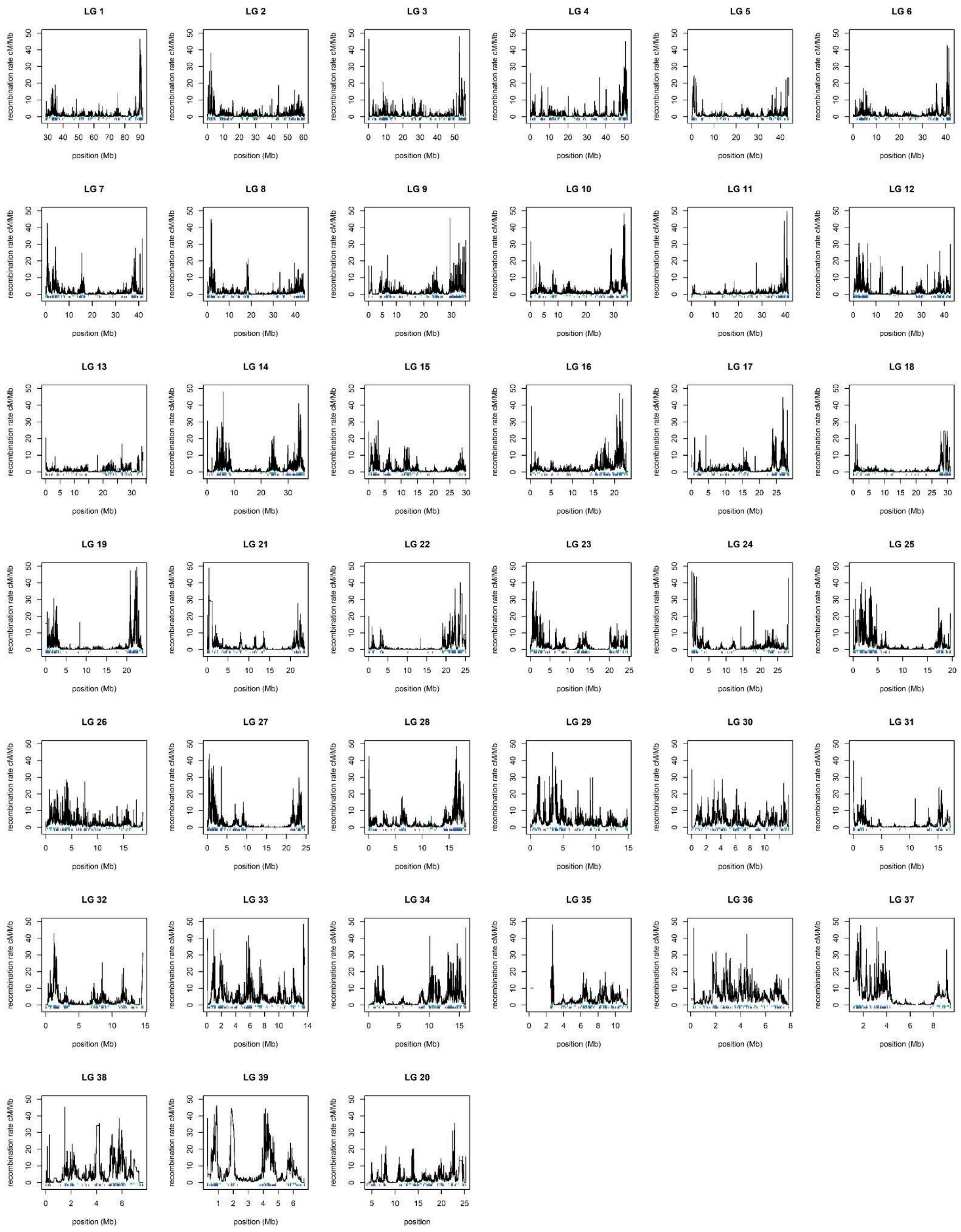

Figure S9. Pyrho maps for Switzerland.

Recombination rates along the physical sequence in the full Swiss dataset (CH - n=76) for all linkage groups in 10 kb windows. Lines below signify male (green) and female (blue) crossover positions.

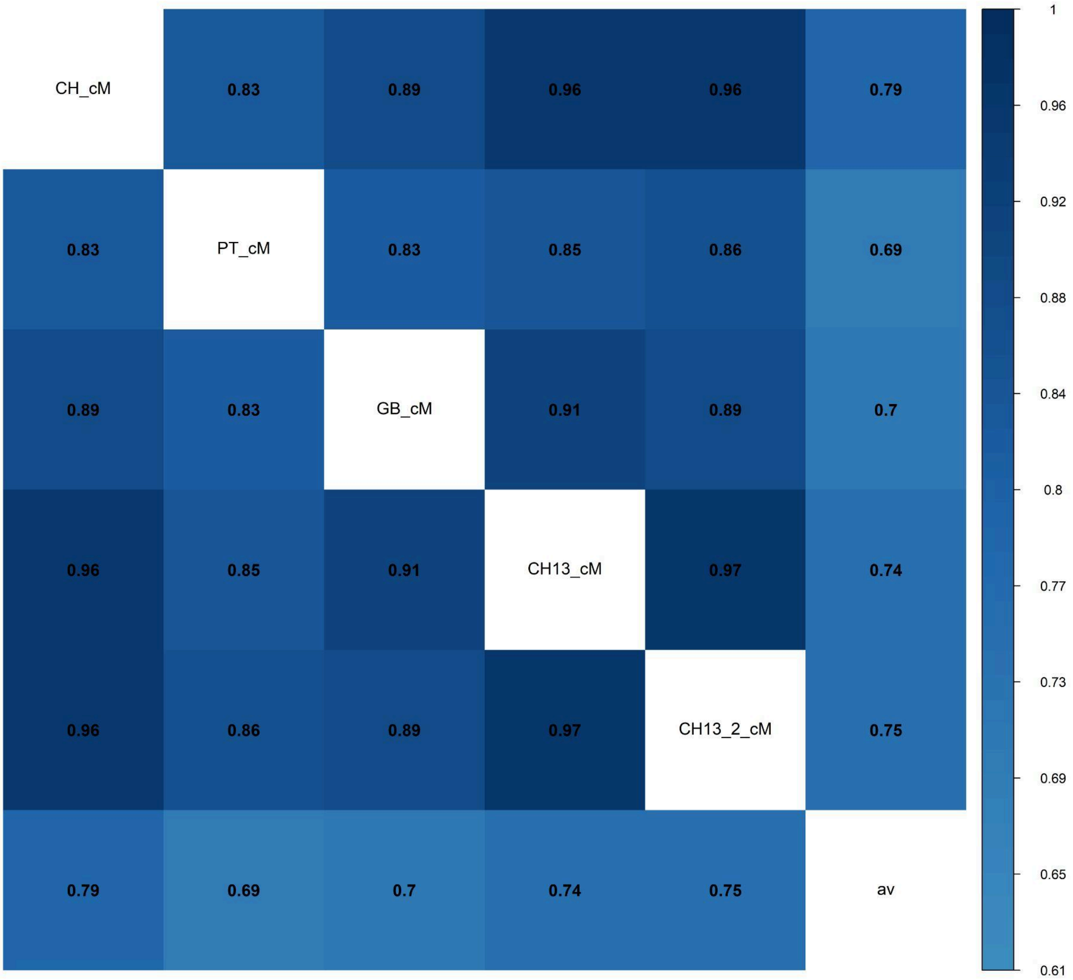

Figure S10. Correlation of linkage map with pyrho results for all populations
Correlation plot among all recombination landscapes inferred from pyrho and sex-averaged estimates through linkage mapping in 1Mb windows. CH: full Swiss dataset (n=76), PT: Portuguese dataset (n=13), GB: Great Britain dataset (n=13), CH13: undersampled first Swiss dataset (n=13), CH13_2: undersampled second Swiss dataset (n=13), av: linkage mapping sex-averaged dataset

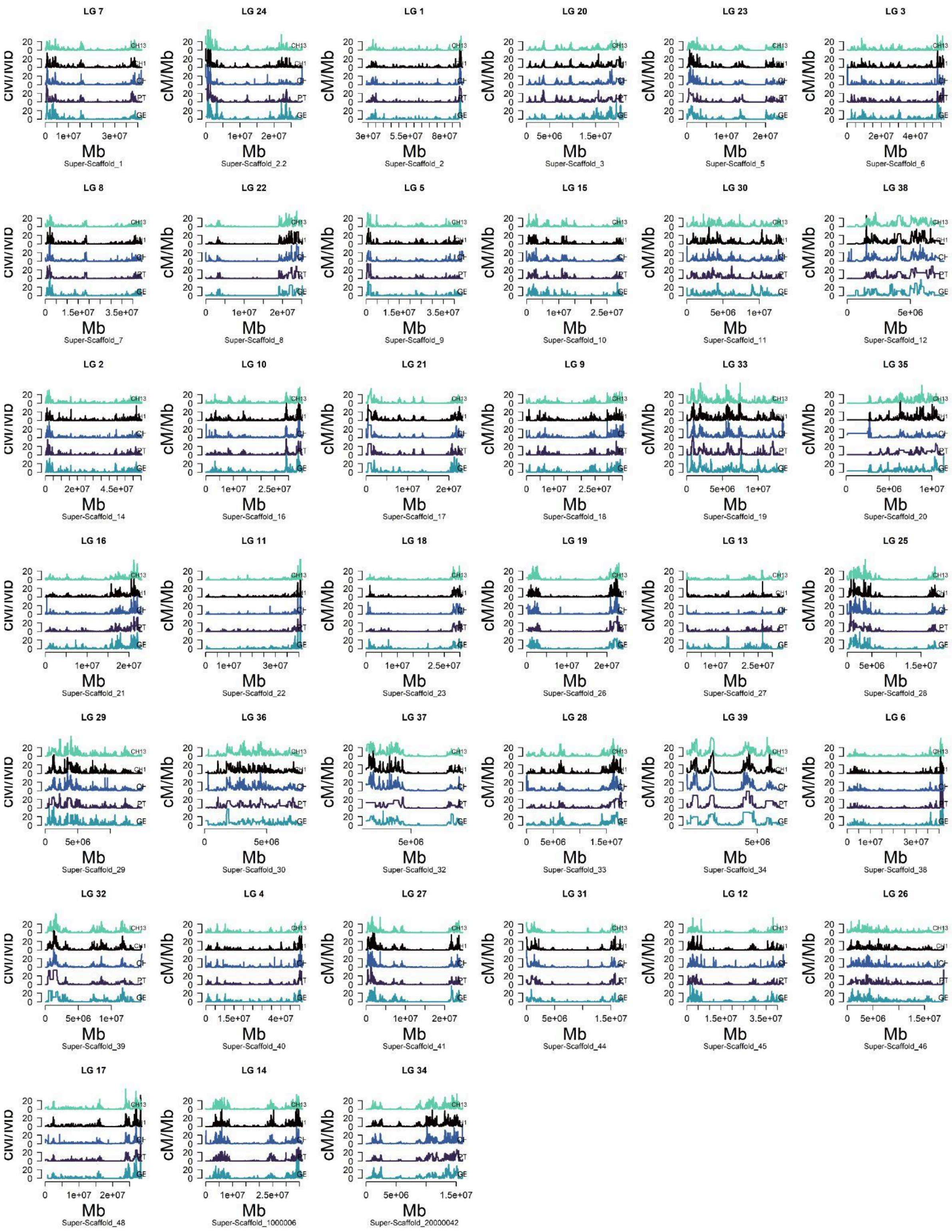

Figure S11. Pyrho results for all populations in 10kb scale

All population recombination rates as the example in Figure 4B in main text plotted in 10kb windows. In each plot populations from top to bottom are CH13_2 (2nd subset of n=13 from Switzerland), CH13 (1st subset of n=13 from Switzerland), CH (full Swiss dataset), PT (Portugal), GB (Great Britain).
